# Ceftazidime is a potential drug to inhibit SARS-CoV-2 infection in vitro by blocking spike protein–ACE2 interaction

**DOI:** 10.1038/s41392-021-00619-y

**Published:** 2021-05-18

**Authors:** ChangDong Lin, Yue Li, YueBin Zhang, ZhaoYuan Liu, Xia Mu, Chenjian Gu, Jing Liu, Yutang Li, GuoHui Li, JianFeng Chen

**Affiliations:** 1 State Key Laboratory of Cell Biology, Center for Excellence in Molecular Cell Science, Shanghai Institute of Biochemistry and Cell Biology, Chinese Academy of Sciences, University of Chinese Academy of Sciences, Shanghai, China; 2 State Key Laboratory of Molecular Reaction Dynamics, Dalian Institute of Chemical Physics, Chinese Academy of Sciences, Dalian, China; 3 Key Laboratory of Medical Molecular Virology (MOE/NHC/CAMS), Department of Medical Microbiology and Parasitology, School of Basic Medical Sciences, Shanghai Medical College, Fudan University, Shanghai, China; 4 BSL-3 laboratory of Fudan University, School of Basic Medical Sciences, Shanghai Medical College, Fudan University, Shanghai, China; 5 School of Life Science, Hangzhou Institute for Advanced Study, University of Chinese Academy of Sciences, Hangzhou, China

**Keywords:** Drug screening, Structural biology, Infection

**Dear Editor**,

Coronavirus disease 2019 (COVID-19) has spread globally as a severe pandemic, which is caused by a novel coronavirus named Severe Acute Respiratory Syndrome coronavirus 2 (SARS-CoV-2).^1^ COVID-19 is a serious threat to healthcare systems, economies, and is devastating to some populations, such as the elderly and those with comorbidities. It is likely to coexist with human beings for a long time. Unfortunately, there is still no effective cure for COVID-19, especially the critically ill patients.

Spike (S) protein on the surface of SARS-CoV-2 facilitates viral entry into target cells by mediating virus receptor recognition and membrane fusion, which contains an S1 subunit at the N terminus and an S2 subunit at the C-terminus. The receptor-binding domain (RBD) at the C-terminus of the S1 subunit has an essential role in mediating viral cell entry through binding to angiotensin-converting enzyme 2 (ACE2) on the plasma membrane of host cells.^2^ Therefore, blocking the binding of spike protein to ACE2 is an effective way to inhibit the infection of target cells by SARS-CoV-2. By now, several studies have reported the development of monoclonal antibodies targeting spike protein, however, the typical timeline for approval of a novel antibody for the management of viral infection is long. In addition, the side effects such as antibody-dependent enhancement of viral infection need to be considered,^3^ and the high cost of antibody treatment will limit the clinical application. Therefore, repurposing of known small molecule drugs to inhibit spike protein and ACE2 binding could significantly accelerate the deployment of effective and affordable therapies for COVID-19.

In order to screen small molecules that block S protein-ACE2 binding, we firstly established an AlphaScreen-based high-throughput system to detect the interaction between S-RBD and extracellular domain (ECD) of ACE2 (Fig. 1a). S-RBD and ACE2-ECD were expressed in 293T cells and then purified. Biotinylated ACE2-ECD (ACE2-ECD-Biotin) binds to streptavidin-coated AlphaScreen donor beads and His-tagged S-RBD (S-RBD-His) binds to anti-His-conjugated AlphaScreen acceptor beads. When S-RBD binds to ACE2-ECD, the two beads come into close proximity. Upon illumination at 680 nm, the donor beads generate singlet oxygen molecules that diffuse to acceptor beads and transfer energy to thioxene derivatives in the acceptor beads resulting in light emission at 520–620 nm. The results showed that the incubation of ACE2-ECD-Biotin with S-RBD-His produced a very strong AlphaScreen signal, and the signal decreased to the basal level in the absence of either of the two proteins (Fig. 1b). To confirm the specificity of this AlphaScreen system, we replaced S-RBD-His with His-tagged extracellular domains of other membrane proteins, including mucosal vascular addressin cell adhesion molecule 1 (MAdCAM-1) and vascular cell adhesion molecule 1 (VCAM-1). Co-incubation of MAdCAM-1-His or VCAM-1-His with ACE2-ECD-Biotin did not generate AlphaScreen signal, indicating that the system detects S-RBD–ACE2 interaction specifically (Fig. 1c).

**Fig. 1:**
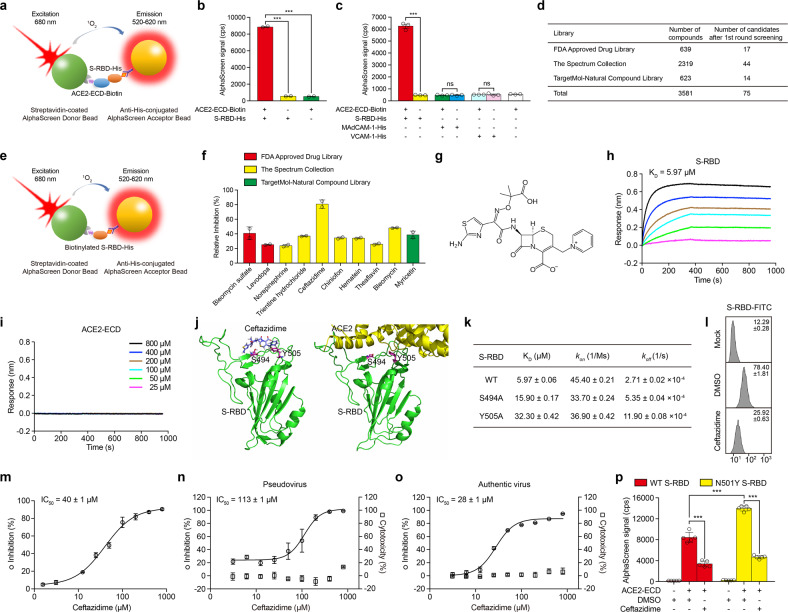
Inhibition of SARS-CoV-2 infection in vitro by ceftazidime. **a** Schematic diagram of AlphaScreen system to detect the interaction between S-RBD and ACE2-ECD, adapted from PerkinElmer application notes. The donor and acceptor beads are coated with streptavidin and anti-His monoclonal antibody, respectively. **b** The interaction between S-RBD and ACE2-ECD was monitored using AlphaScreen system. **c** Comparison of the AlphaScreen signal of S-RBD-His, MAdCAM-1-His and VCAM-1-His proteins in the presence or not of ACE2-ECD-Biotin in AlphaScreen system. **d** Libraries used in AlphaScreen-based high-throughput system and 75 candidates were identified from 3581 compounds in positive selection. The inhibition rate was calculated by the decrease of AlphaScreen signal of each compound compared with that of DMSO vehicle control group. All the compounds showed over 45% inhibition rate were defined as positive candidates. **e** Schematic diagram of negative selection using AlphaScreen system, adapted from PerkinElmer application notes. Biotinylated S-RBD-His simultaneously links streptavidin-coated donor bead and anti-His-conjugated acceptor bead together to generate AlphaScreen signal directly. **f** Relative inhibition of 10 candidate compounds on S-RBD–ACE2 interaction using AlphaScreen system. The relative inhibition rate was calculated by subtracting the inhibition rate in negative selection from that in positive selection. **g** Molecular structure of ceftazidime. Binding of ceftazidime to S-RBD (**h**) or ACE2-ECD (**i**) was measured by BLI experiments in an Octet RED96 instrument. The biotin-conjugated S-RBD or ACE2-ECD was captured by streptavidin that was immobilized on a biosensor and tested for binding with gradient concentrations of ceftazidime. **j** Snapshot of the lowest energy binding pose of ceftazidime–S-RBD based on MD simulation (left) and the crystal structure of S-RBD and ACE2 complex (PDB code 6M0J) (right). **k** Binding capacity of ceftazidime to WT and mutated S-RBD. The values of K_D_, *k*_on_ and *k*_off_ for the binding of ceftazidime to WT and mutated S-RBD (S494A and Y505A) were obtained by BLI experiments (K_D_ = *k*_off_/*k*_on_). **l** Soluble S-RBD binding to HPAEpiC cells was examined by flow cytometry analysis. Mock, cells were incubated with FITC-conjugated goat anti-human IgG; DMSO, vehicle control; Ceftazidime, 100 μM ceftazidime in DMSO. Numbers within the panel showed the specific mean fluorescence intensities. **m** The inhibitory effect of ceftazidime on the binding of S-RBD protein to HPAEpiC cells. Cells were treated with different concentrations of ceftazidime. The inhibition rate was calculated by the decrease of the mean fluorescence intensity of each group compared with that of DMSO vehicle control group. IC_50_ was indicated in the graph. **n**, **o** Cells were treated with DMSO or serially diluted ceftazidime. Inhibition of luciferase-encoding SARS-CoV-2 typed pseudovirus entry into ACE2-expressing 293T cells by ceftazidime (**n**). Inhibition of authentic SARS-CoV-2 infection of Vero E6 cells by ceftazidime (**o**). IC_50_ was indicated in the graph. The cell viability was assessed by Cell Counting Kit-8 (CCK-8) assay and the cytotoxicity (%) of ceftazidime to 293T and Vero E6 cells was calculated by the decrease of optical density (OD) at each concentration of ceftazidime compared with that of DMSO vehicle control. **p** Effect of ceftazidime on WT/N501Y S-RBD–ACE2 interaction indicated by AlphaScreen signal. DMSO, vehicle control. One representative result of three independent experiments is shown in (**h**), (**i**) and (**l**). Data represent the mean±SEM (*n* ≥ 2) in (**b**), (**c**), (**f**) and (**k**)–(**p**). ***p < 0.001; ns not significant (unpaired two-tailed Student’s *t* test)

Next, we used the AlphaScreen-based high-throughput system to screen small molecules that block S-RBD–ACE2 interaction. A total of 3581 small molecule compounds with known molecular structures from FDA Approved Drug Library, Spectrum Collection, and TargetMol-Natural Compound Library were assessed (Fig. 1d). The assay was conducted at a final compound concentration of 10 μM and the interaction between S-RBD-His (0.1 μM) and ACE2-ECD-Biotin (0.2 μM) was analyzed. After the first-round screening, 75 candidate compounds were identified, which showed an inhibitory effect on S-RBD–ACE2 interaction (Fig. 1d). All these compounds showed over 45% inhibition rate according to the changes in AlphaScreen signal. To exclude the interference of the compounds to the AlphaScreen system per se, we designed a negative selection system in which the biotinylated S-RBD-His links streptavidin-coated AlphaScreen donor bead and anti-His-conjugated AlphaScreen acceptor bead together to generate AlphaScreen signal directly (Fig. 1e). After the negative selection, 10 compounds, including bleomycin sulfate, levodopa, norepinephrine, trientine hydrochloride, ceftazidime, chiniofon, hematein, theaflavin, bleomycin and myricetin, from the 75 candidate compounds were validated to inhibit S-RBD–ACE2 interaction effectively. Among these ten compounds, ceftazidime was the most potent inhibitor which showed a relative inhibition rate of about 80% (Fig. 1f). Thus, ceftazidime was selected for further investigation considering the best inhibitory effect on S-RBD–ACE2 interaction, the anti-inflammatory effect, and the minimal side effect of this drug compared with the other nine compounds^4^ (Fig. 1g).

To investigate whether S-RBD or ACE2 is the binding target protein of ceftazidime, we applied a bio-layer interferometry (BLI) experiment to examine the binding affinity between ceftazidime and S-RBD or ACE2-ECD. Along with the elevated concentration of ceftazidime, this compound showed increased binding to S-RBD protein with an K_D_ value of 5.97 μM (Fig. 1h). Notably, ceftazidime had a slow dissociation rate from S-RBD (2.71 ± 0.02 × 10^−4^ s^−1^) (Fig. 1k), indicating a strong and stable interaction between ceftazidime and S-RBD. By contrast, ceftazidime and ACE2-ECD showed no specific binding signal (Fig. 1i). Thus, ceftazidime binds to S-RBD specifically.

In order to further understand the mechanism of ceftazidime binding to S-RBD, all-atom molecular dynamics (MD) simulations with multiple walkers metadynamics were used to explore the possible binding sites of ceftazidime in S-RBD.^5^ Initially, five copies of ceftazidime molecule were randomly inserted around S-RBD within a rectangular box (8 nm × 8 nm × 10 nm) to enhance the binding process, yielding a stoichiometric ratio of 5:1 (ceftazidime:S-RBD) in the MD simulation system. Moreover, the coordination numbers between each individual ceftazidime molecule and S-RBD were used as collective variables to drive the binding in the well-tempered metadynamic simulations, and eight replicas (walkers) were employed to parallelize the simulations. After 500 ns metadynamic simulation of each replica, the average spatial density distributions of ceftazidime molecules were analyzed, and the lowest energy binding pose was shown (Fig. 1j). After extracting MD trajectories at this binding site and performing MM/GBSA analysis to identify the key residues responsible for ceftazidime binding, we found that residues Ser494 (S494) and Tyr505 (Y505) at the ACE2 binding interface in S-RBD played key roles in stabilizing ceftazidime binding. Computational alanine scanning results indicated that S494A and Y505A would weaken the binding energy of ceftazidime by 1.97 kcal/mol and 3.28 kcal/mol, respectively. To verify whether S494 and Y505 were indeed hotspots responsible for ceftazidime binding, we constructed two single-point mutants of S-RBD (S494A and Y505A) experimentally and performed BLI experiments to examine the binding affinities between ceftazidime and the two S-RBD mutants. In consistent with the MD simulation results, both S494A and Y505A S-RBD mutants exhibited significantly faster dissociation (*k*_off_) rates and increased K_D_ values compared with WT S-RBD (Fig. 1k). These data indicated that ceftazidime and ACE2 share the same binding interface in S-RBD. Thus, the binding of ceftazidime at this interface will block ACE2 binding to S-RBD.

The lung is the main organ infected by SARS-CoV-2, which causes severe acute respiratory syndrome (SARS). Therefore, we examined the inhibitory effect of ceftazidime on the binding of S-RBD protein to human pulmonary alveolar epithelial cells (HPAEpiC), which express ACE2. The addition of 100 μM ceftazidime into the soluble S-RBD binding assay led to a significant decrease in the S-RBD binding signal (Fig. 1l), demonstrating the efficient inhibition on S-RBD binding to HPAEpiC cells by ceftazidime. Further analysis showed a half-maximal inhibitory concentration (IC_50_) of 40 ± 1 μM (Fig. 1m).

To evaluate the inhibitory effect of ceftazidime on the entry of SARS-CoV-2 pseudovirus into 293T cells overexpressing human ACE2, we added ceftazidime into the SARS-CoV-2 pseudovirus infection assay at a series of concentrations. The results showed that ceftazidime efficiently inhibited SARS-CoV-2 pseudovirus cell entry in vitro and the IC_50_ was 113 ± 1 μM (Fig. 1n).

Next, we examined the inhibition of the authentic SARS-CoV-2 infection of Vero E6 cells by ceftazidime. SARS-CoV-2 was pre-mixed with DMSO or ceftazidime at a series of concentrations at 37 °C for 1 h, and then cocultured with Vero E6 cells at 37 °C for another 1 h. Unbound SARS-CoV-2 virions were removed by washing cells with PBS, followed by culturing with fresh medium containing ceftazidime at the same concentration as in the pre-mixture of SARS-CoV-2. At 48 h post infection, ceftazidime showed apparent viral inhibition at the concentration above 25 μM (Supplementary Fig. S1). Moreover, viral RNA level in the culture supernatant was quantified with qRT-PCR and the inhibition rate was calculated. The results showed that ceftazidime strongly inhibited the infection of Vero E6 cells by SARS-CoV-2 virions with an IC_50_ of 28 ± 1 μM (Fig. 1o). Of note, ceftazidime showed negligible cytotoxicity even at high concentrations, indicating its safety for clinical usage (Fig. 1n and o).

Recently emerged SARS-CoV-2 variants carry the N501Y mutation in S-RBD. To further investigate the effect of ceftazidime on the binding of S-RBD N501Y mutant to ACE2, we purified N501Y S-RBD protein and performed the AlphaScreen assay. The results showed that the AlphaScreen signal of N501Y S-RBD–ACE2 was significantly higher than that of WT S-RBD–ACE2, which was consistent with the results that the N501Y mutation markedly increase affinity for ACE2 reported in other studies. Of note, ceftazidime still showed a strong inhibition on N501Y S-RBD–ACE2 interaction (Fig. 1p). Thus, ceftazidime can inhibit the binding of both WT S-RBD and N501Y mutant to ACE2.

Ceftazidime has been clinically used as a drug for the treatment of bacterial pneumonia and the blood concentration of ceftazidime can reach over 300 μM. At this concentration, ceftazidime showed an approximate 95% inhibition of SARS-CoV-2 pseudovirus infection and 85% inhibition of authentic SARS-CoV-2 infection *i*n vitro, indicating its strong potency to inhibit cell entry of SARS-CoV-2. The binding site identified based on our MD simulation results for ceftazidime on S-RBD overlaps with that of ACE2. Especially, ceftazidime would occupy the crucial site Y505 via strong π–π interaction, where a single-point mutation Y505A was found to be sufficient for completely abolishing the binding of ACE2. Therefore, the occupation of this interface in S-RBD by ceftazidime could block the binding of ACE2, leading to the consequent inhibition of cell entry of SARS-CoV-2.

Cephalosporins have many derivatives which share the similar core structure but have different side-chain modifications. We have also compared the inhibitory effects of 14 different cephalosporins, including ceftazidime, cephradine, cefazolin, cephalexin, cefuroxime, cefamandole, cefuroxime axetil, cefotaxime, ceftriaxone, cefoperazone, cefoselis, cefepime, ceftobiprole and ceftaroline. Among all cephalosporins, only ceftazidime showed a strong inhibition on the S-RBD–ACE2 interaction. Ceftobiprole and ceftriaxone showed limited inhibitory effects, whereas other cephalosporins have little or no inhibitory effect on S-RBD–ACE2 binding (Supplementary Fig. S2). These results in combination with a preliminary Structure Activity Relationship analysis suggested that the unique moieties in ceftazidime, including 2-aminothiazole, oxime protected with a terminal-exposed isobutyric acid and the positive charged pyridine, might be involved in mediating the binding to S-RBD and eventually blocked the protein interaction between S-RBD and ACE2.

Although ceftazidime showed a promising inhibitory effect on SARS-CoV-2 infection of cells in vitro, there are some potential limitations when used clinically. First, ceftazidime is a β-lactam antibiotic which is easily degraded by β-lactamase in vivo. A combination usage of β-lactamase inhibitor may reduce this limitation. Second, ceftazidime might not be used clinically for the prevention of SARS-CoV-2 infection to avoid the induction of bacterial drug resistance. Moreover, the working concentration of ceftazidime for inhibiting SARS-CoV-2 infection of cells in vitro is relatively high, therefore further optimization of ceftazidime structure to improve its binding affinity to S-RBD need to be further explored.

In summary, we identified ceftazidime as a potential drug to inhibit SARS-CoV-2 infection in vitro by binding to S-RBD and consequently blocking S-RBD interaction with ACE2. Since ceftazidime is a drug clinically used for the treatment of pneumonia with affordable price and minimal side effects compared with other antiviral drugs, ceftazidime should be considered as one of the first-line antibiotics used for the treatment of COVID-19, which deserves the immediate preclinical and clinical trials.

## Supplementary information

Supplementary materials
