# Correction To: Ceftazidime is a potential drug to inhibit SARS-CoV-2 infection in vitro by blocking spike protein–ACE2 interaction

**DOI:** 10.1038/s41392-021-00643-y

**Published:** 2021-06-10

**Authors:** ChangDong Lin, Yue Li, YueBin Zhang, ZhaoYuan Liu, Xia Mu, Chenjian Gu, Jing Liu, Yutang Li, GuoHui Li, JianFeng Chen

**Affiliations:** 1 State Key Laboratory of Cell Biology, Center for Excellence in Molecular Cell Science, Shanghai Institute of Biochemistry and Cell Biology, Chinese Academy of Sciences, University of Chinese Academy of Sciences, Shanghai, China; 2 State Key Laboratory of Molecular Reaction Dynamics, Dalian Institute of Chemical Physics, Chinese Academy of Sciences, Dalian, China; 3 Key Laboratory of Medical Molecular Virology (MOE/NHC/CAMS), Department of Medical Microbiology and Parasitology, School of Basic Medical Sciences, Shanghai Medical College, Fudan University, Shanghai, China; 4 BSL-3 laboratory of Fudan University, School of Basic Medical Sciences, Shanghai Medical College, Fudan University, Shanghai, China; 5 School of Life Science, Hangzhou Institute for Advanced Study, University of Chinese Academy of Sciences, Hangzhou, China

**Keywords:** Drug screening, Structural biology, Infection

Correction to: *Signal Transduction and Targeted Therapy* 10.1038/s41392-021-00619-y, published online 18 May 2021

After online publication of the article^1^ we noticed an error in citations for affiliation in the supplementary materials published in this article.

The citations for affiliation in the article were reordered during the production process to match the order of authors in the author group. Unfortunately, this was not reflected in the supplementary material.

We regret for not processing the revised supplementary material during the production process. Corrected supplementary material is published with this correction note.

The original article has been corrected.

## Supplementary information

Supplementary Materials
