# Supplementary material for: Ceftazidime is a potential drug to inhibit SARS-CoV-2 infection in vitro by blocking spike protein–ACE2 interaction
Source: Signal Transduct Target Ther. 2021 May 18;6:198. doi: 10.1038/s41392-021-00619-y (PMC8129692; doi:10.1038/s41392-021-00619-y)
Supplement: Supplementary file 1 — Supplementary materials [file 41392_2021_619_MOESM1_ESM.docx]

Supplementary Materials for

**Ceftazidime is a potential drug to inhibit SARS-CoV-2 infection in vitro by blocking spike protein-ACE2 interaction**

ChangDong Lin, Yue Li, YueBin Zhang, ZhaoYuan Liu, Xia Mu, Chenjian Gu, Jing Liu, Yutang Li, GuoHui Li, and JianFeng Chen

Correspondence to: jfchen@sibcb.ac.cn (J.F.C.), ghli@dicp.ac.cn (G.H.L.)

**This PDF file includes:**

Materials and Methods

Supplementary Fig. S1 to S2

**Materials and Methods**

**Cell lines and compounds**

Human embryonic kidney HEK293T cells and African green monkey kidney Vero E6 cells were cultured at 37°C with 5% CO_2_ in Dulbecco’s Modified Eagle Medium (DMEM) (Gibco, Carlsbad, CA, USA) containing 2 mM L-glutamine, 100 U/ml penicillin, 100 μg/ml streptomycin, and 10% (vol/vol) fetal bovine serum (Gibco, Carlsbad, CA, USA). HPAEpiC cells were cultured at 37°C with 5% CO_2_ in Alveolar Epithelial Cell Medium (ScienCell, Carlsbad, CA, USA) containing 2 mM L-glutamine, 100 U/ml penicillin, 100 μg/ml streptomycin, and 10% (vol/vol) fetal bovine serum (Gibco, Carlsbad, CA, USA).

FDA Approved Drug Library, Spectrum Collection and TargetMol-Natural Compound Library were from Chemical Biology Core Facility in Center for Excellence in Molecular Cell Science, Shanghai Institute of Biochemistry and Cell Biology, Chinese Academy of Sciences. Ceftazidime was purchased from MedChemExpress (NJ, USA).

**Protein expression and purification**

Recombinant SARS-CoV-2 S-RBD fused with Fc/His tag (S-RBD-His) was expressed in 293T cells, and isolated by affinity chromatography using Protein A Agarose (Thermo Fisher Scientific, MA, USA). Recombinant human ACE2-ECD fused with Flag tag was produced in 293T cells and purified by affinity chromatography using anti-DYKDDDDK G1 Resin according to the manufacturer’s instructions (GenScript, NanJing, China). Purified proteins were analyzed by SDS-PAGE to ensure purity and appropriate molecular weights.

**AlphaScreen**

AlphaScreen assays were performed in Costar 384-well microplates in a 20 μl final reaction volume. Streptavidin-coated AlphaScreen donor beads or anti-His-conjugated AlphaScreen acceptor beads (PerkinElmer, Waltham, MA, USA) were used. The ACE2-ECD protein was labeled with biotin (10 mM) in advance, according to the manufacturer’s instructions (PerkinElmer, Waltham, MA, USA). The assays were performed in PBS buffer (155 mM NaCl, 1.06 mM KH_2_PO_4_, 2.97 mM Na_2_HPO_4_, pH 7.4) and 0.1% BSA. 5 μl S-RBD-His (final concentration 0.1 μM) and 5 μl ACE2-ECD-Biotin (final concentration 0.2 μM) were pre-incubated with compounds at a final concentration of 10 μM for 0.5 h at 4 °C. Then donor beads and acceptor beads (final concentration 10 μg/ml) were added into the reaction in dark for 2 h at room temperature. Laser excitation was carried out at 680 nm, and readings were performed at 520 to 620 nm using the EnVision (PerkinElmer, Waltham, MA, USA) plate reader.

**Bio-layer Interferometry (BLI) Experiment**

The BLI experiment was performed using an Octet Red96 instrument (ForteBio, Fremont, CA, USA). Briefly, biotinylated S-RBD or ACE2-ECD (50 μg/ml) was immobilized on streptavidin (SA) biosensors and then incubated with gradient concentrations of ceftazidime in kinetics buffer (PBS and 0.02% Tween-20). The association and dissociation steps were set to 360 s and 600 s. The K_D_ value of WT S-RBD binding affinity for ceftazidime was calculated from all the binding curves based on their global fit to a 1:1 Langmuir binding model with an R^2^ value of ≥ 0.95. The K_D_ values of the two S-RBD mutants (S494A and Y505A) binding affinity for ceftazidime were calculated from the curves based on their local fit with 200 μM ceftazidime. The kinetically derived affinities were calculated as K_D_ = *k*_off_ / *k*_on_. Binding experiments were performed at 25 °C. Data were analyzed using Octet Data Analysis Software 9.0 (ForteBio, Fremont, CA, USA).

**Molecular dynamics (MD) simulations**

The initial conformation of SARS-CoV-2 S-RBD was obtained from the crystal structure of S-RBD and ACE2 complex (PDB code 6M0J). S-RBD was first centered into a rectangular box (8 nm × 8 nm × 10 nm) and five copies of ceftazidime molecule were randomly inserted around S-RBD. The simulation system was set up using Amber Tools 19 and the AMBER ff14SB force field was used for protein and the General Amber Force Field (GAFF) was used for ceftazidime. The Gaussian 09 package with B3LYP/6-31G* method was employed to perform the geometry optimization of ceftazidime and the restrained electrostatic potential (RESP) approach was used to assign the partial charge of ceftazidime. The TIP4PEW water model was used to solvate the system and NaCl ions were used to neutralize the charges. S-RBD contains 4 disulfide bonds ^1^ and two sites (N331 and N343) of S-RBD were glycosylated ^2,3^. The system was initially refined using 500 steepest descent steps before switching to conjugate gradient energy minimization and gradually heated to 300 K within 2 ns. The positional restraints were exerted on S-RBD with a weight of 10 kcal/mol/Å2 during the energy minimization and heating process. Then the restraints were released within six equilibration steps in the NPT ensemble. The hydrogen mass repartitioning was set to 4 amu to enable an integration step of 4 fs for the simulations.

The multiple walkers metadynamic simulations were performed using OpenMM package through GPU acceleration combining with PLUMED enhanced-sampling library ^4^. The Langevin Integrator was used with a collision frequency of 1.0 ps-1 to couple the system’s temperature at 300 K. The Particle-Mesh Ewald (PME) and the dispersion correction algorithm were exploited to estimate the contribution of long-range non-bonded interactions that beyond the cutoff of 12 Å. The coordination numbers between each individual ceftazidime molecule and S-RBD were used as collective variables (CVs) to drive the binding in the well-tempered metadynamic simulations and 8 replicas (walkers) were employed to parallelize the simulations. The switching distance for estimating the coordination numbers were set at 2.5 Å and the neighbor list was used to speed up the calculation with an updating frequency of 100 steps. For each well-tempered metadynamic simulation, a biasfactor of 30 was used and the Gaussian widths were set to 1.0 with the height of 2.0 KJ/mol added by every 100 ps. The average spatial density distributions of ceftazidime were analyzed using volmap command in VMD. The per-residue decomposition calculation and computational alanine scanning were performed using MMPBSA.py module in AMBER package.

**Flow cytometry**

0.1 μM S-RBD-Fc/His was pre-incubated with 5 μg/ml FITC-conjugated goat anti-human IgG in 50 μL of PBS and then incubated with HPAEpiC cells for 30 min at room temperature. Cells were washed twice before flow cytometry analysis. Cells were incubated with FITC-conjugated goat anti-human IgG merely as a control.

**Pseudotyped SARS-CoV-2 infection assay**

SARS-CoV-2 pseudoviruses were produced as previously described ^5^. The pseudoviruses were diluted in complete DMEM mixed with an equal volume (50 μl) of diluted DMSO or ceftazidime, and then incubated at 37 °C for 1 h. The mixture was incubated with 293T cells stably expressing human ACE2 at 37 °C for 12 h, followed by change of fresh medium containing ceftazidime of the corresponding concentration. At 36 h post incubation, the cells were lysed with Bio-Lite Luciferase Assay Buffer and tested for luciferase activity (Vazyme, Nanjing, China). The percent neutralization was calculated by comparing the luciferase value of ceftazidime treatment group to that of DMSO vehicle control.

**Authentic SARS-CoV-2 infection assay**

For evaluation of antiviral effect of ceftazidime, Vero E6 cells were seeded in 96-well plates at 1 day prior to infection. DMSO or serially diluted ceftazidime was mixed with SARS-CoV-2 (GenBank: MT121215.1) at 100 TCID50 per well and incubated at 37 °C for 1 h. Then Vero E6 cells were incubated with DMSO/ceftazidime-virus mixture at 37 °C for 1 h. Unbound SARS-CoV-2 virions were removed by washing cells with PBS, followed by culturing with fresh medium containing ceftazidime of the corresponding concentration. At 48 h post infection, all wells were examined for the cytopathic effect (CPE) and the culture supernatants were collected for viral RNA detection. Viral RNA was extracted using TRIzol LS reagent (Invitrogen, Carlsbad, CA, USA) following the manufacturer’s instructions. The viral nucleocapsid gene-based quantification assay was developed using the Verso 1-step RT-qPCR Kit (Thermo Fisher Scientific, Waltham, MA, USA) on CFX96™ Real-Time PCR System (Bio-Rad, Hercules, CA, USA). The PCR primers targeting the N gene (nt608-706) of SARS-CoV-2 were: 5’-GGGGAACTTCTCCTGCTAGAAT-3’/5’-CAGACATTTTGCTCTCAAGCTG-3’ (forward/reverse). The inhibition rate was calculated finally. All the infection experiments were performed in the biosafety level-3 (BSL-3) laboratory of Fudan University.

**Cell cytotoxicity assay**

The Cell Counting Kit-8 (CCK-8, Beyotime, Shanghai, China) was used to assess cell viability according to the manufacturer’s instructions. Briefly, 293T or Vero E6 cells were dispensed into 96-well plate (5.0 × 10^4^ cells in 100 μl medium supplemented with different concentrations of ceftazidime per well) for 48 h. 10 μl CCK-8 reagent was added into the medium for 1 h at 37 °C and then the absorbances at 450 nm were measured using a microplate reader (Thermo Fisher Scientific, Waltham, MA, USA). The cytotoxicity (%) of ceftazidime to 293T and Vero E6 cells was calculated by the decrease of optical density (OD) at each concentration of ceftazidime compared with that of DMSO vehicle control.

**QUANTIFICATION AND STATISTICAL ANALYSIS**

Statistical significance was determined by unpaired two-tailed Student’s *t* test (GraphPad, version 5.01, CA, USA). The resulting *p* values are indicated as follows: ns, not significant; *, *p* < 0.05; **, *p* < 0.01; ***, *p* < 0.001. Data represent the mean ± SEM of at least two independent experiments.

**REFERENCES**

1 Lan, J. *et al.* Structure of the SARS-CoV-2 spike receptor-binding domain bound to the ACE2 receptor. *Nature* **581**, 215-+, doi:10.1038/s41586-020-2180-5 (2020).

2 Li, Q. Q. *et al.* The Impact of Mutations in SARS-CoV-2 Spike on Viral Infectivity and Antigenicity. *Cell* **182**, 1284-+, doi:10.1016/j.cell.2020.07.012 (2020).

3 Zhang, Y. *et al.* Site-specific N-glycosylation Characterization of Recombinant SARS-CoV-2 Spike Proteins. *Mol Cell Proteomics*, doi:10.1074/mcp.RA120.002295 (2020).

4 Raiteri, P., Laio, A., Gervasio, F. L., Micheletti, C. & Parrinello, M. Efficient reconstruction of complex free energy landscapes by multiple walkers metadynamics. *J Phys Chem B* **110**, 3533-3539, doi:10.1021/jp054359r (2006).

5 Ou, X. *et al.* Characterization of spike glycoprotein of SARS-CoV-2 on virus entry and its immune cross-reactivity with SARS-CoV. *Nat Commun* **11**, 1620, doi:10.1038/s41467-020-15562-9 (2020).

**Supplementary Fig. S1**


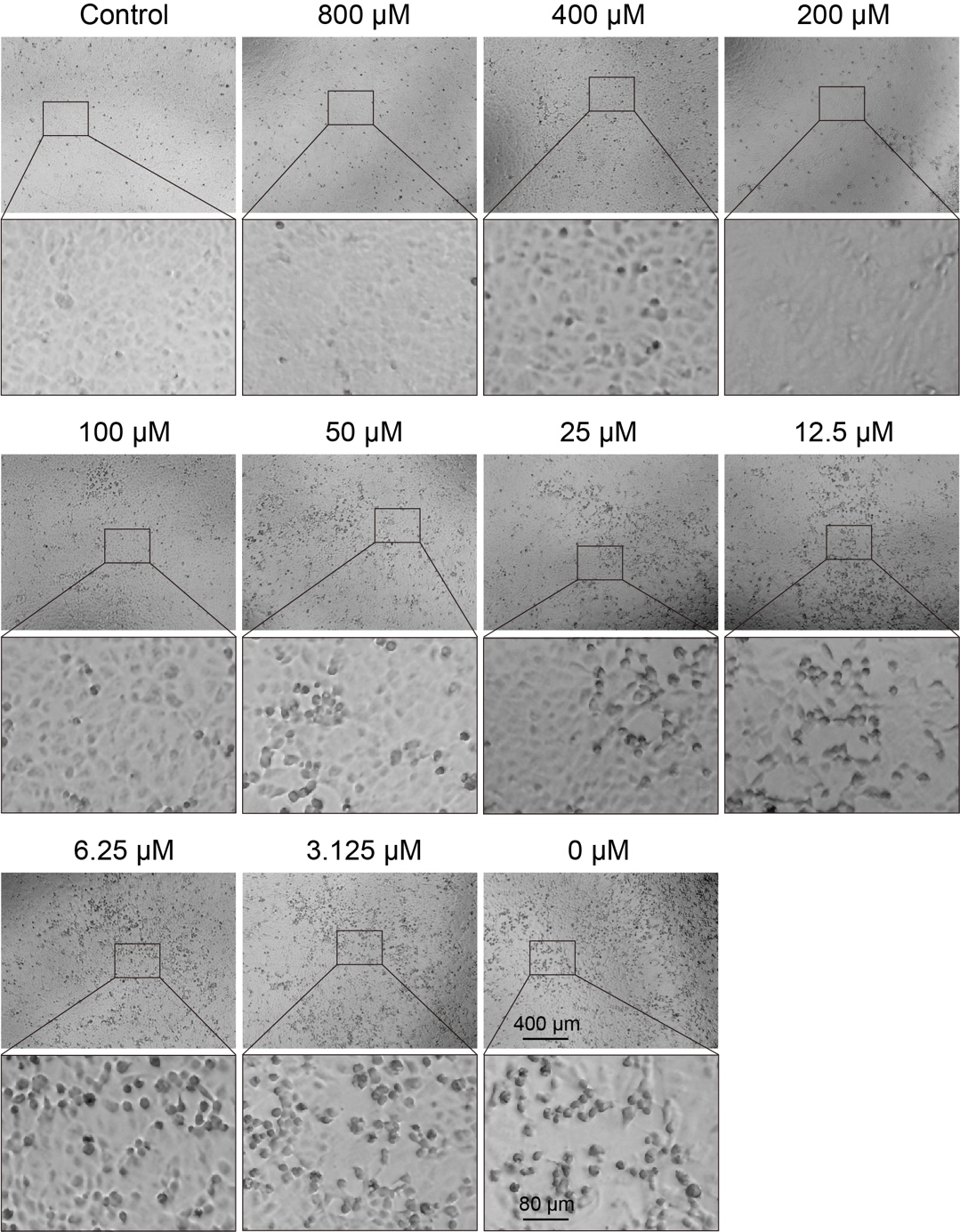


**Supplementary Fig. S1. Inhibition of authentic SARS-CoV-2 infection of Vero E6 cells by ceftazidime.**

Vero E6 Cells were treated with authentic SARS-CoV-2 and DMSO or serially diluted ceftazidime. Phase-contract image of Vero E6 cells examining the cytopathic effect.

One representative result of three independent experiments is shown.

**Supplementary Fig. S2**


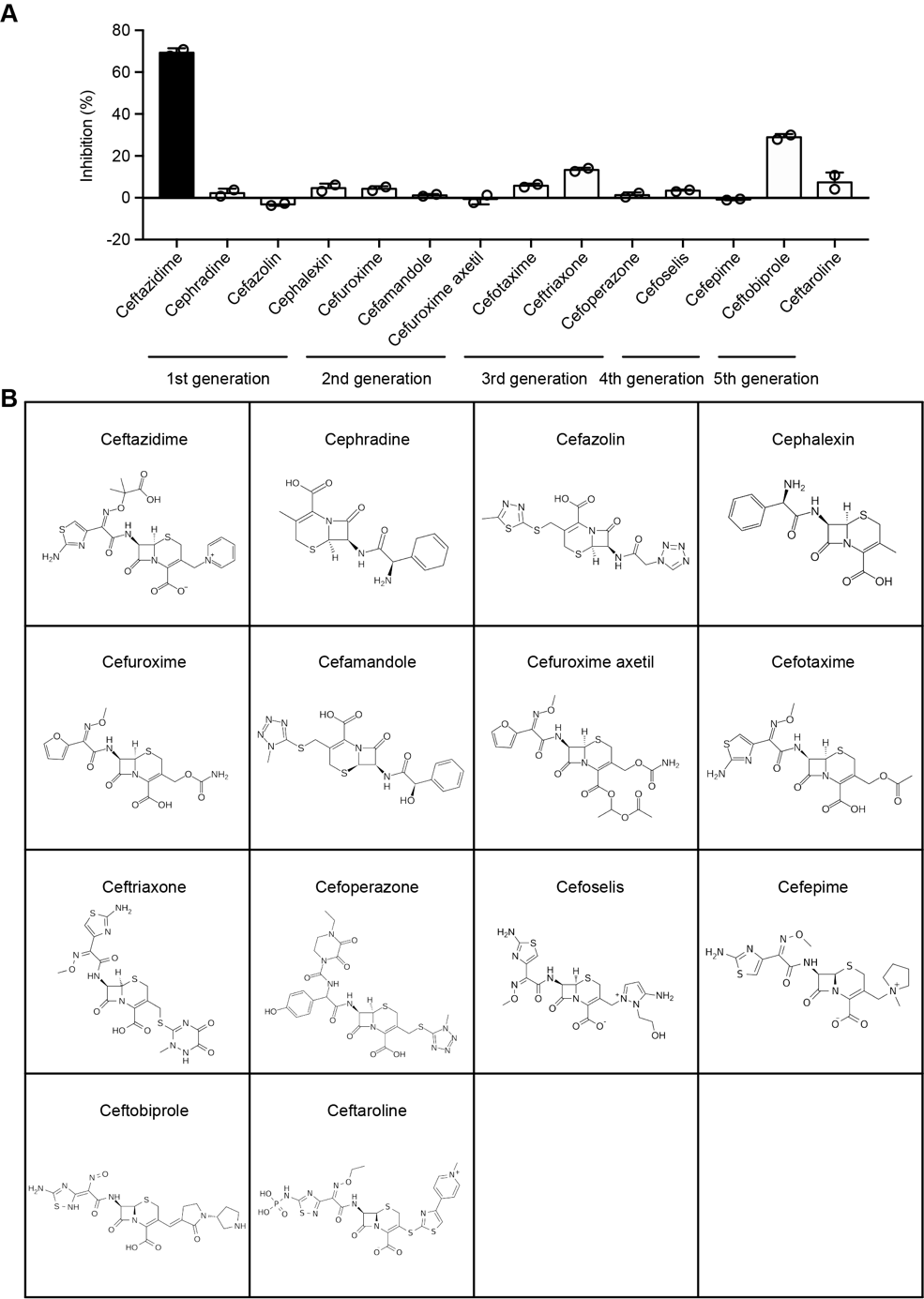


**Supplementary Fig. S2. Effect of ceftazidime and the derivatives of cephalosporins on S-RBD–ACE2 interaction.**

**a** Inhibition of S-RBD–ACE2 interaction by ceftazidime and the derivatives of cephalosporins was analyzed using AlphaScreen system.

**b** Molecular structures of ceftazidime and the derivatives of cephalosporins.
